# 

**Published:** 1816-10

**Authors:** 


### CATALOGUE OF MEDICAL BOOKS.

A TREATISE on Uterine Hæmorrhage; by Duncan Stewart, M.D. Physician-accoucheur to the Westminster General Dispensary, and Lecturer on Midwifery in London. 8vo.

The London Dissector; comprising a Description of the Muscles, Vessels, Nerves, and Viscera of the Human Body, as they appear on Dissection. Fifth edition. 12mo.

A Catalogue of an extensive Collection of Books on Anatomy, Medicine, Surgery, Midwifery, Chemistry, Botany, &c. &c. New and Second Hand; including a Selection of Foreign Medical Works recently imported from the Continent. Sold by John Anderson, Medical Bookseller, West Smithfield, London. To which are added, Tables of the Pay of the Medical Department, of the Army, Navy, and East India Company's Service, a complete List of the Lectures delivered in London, with their terms, hours of attendance, &c. &c. 12mo.

A Letter to the Right Honorable Lord Binning, M.P. &c. &c. containing some Remarks on the State of Lunatic Asylums, and on the Number and Condition of the Insane Poor in Scotland, 8vo.

A Catalogue of Medical Books, containing the most Modern and and approved Works on Anatomy, Medicine, Surgery, Midwifery, Materia Medica, Chemistry, &c. &c.; selling by Highley and Son, 174, Fleet-street. To which is added, a List of all the Lectures delivered in London, with the Terms, Hours of Attendance, &c. the Pay of the Medical Department of the Army, Navy, and East India Company's Service.

---

### *NOTICES TO CORRESPONDENTS.*

Dr. P.'s *Lectures may be announced in general terms in our Intelligence. If he wishes to descend to any particulars, they must be consigned to the Cover, or be stitched with the Number, on the usual terms of advertisements.*

*Communications have reached us from* Mr. GREEN, Mr. MILLAR, Mr. ROSE, CHIRURGUS, *and others.*

Mr. ROSE *must permit us to shorten his Communication, as some of his facts have been accurately noticed during his absence in the service of his country.*

Mr. WOODHOUSE's *Letter will be considered an Advertisement by his Majesty's Commissioners.*

*Two of our Correspondents are informed, that an account of* Mr. WANT's *discovery will be found in various Numbers of Vol.* xxxii. *for the year* 1815, *and the Recipe for the Tincture is at p.* 78. *It is not inserted in the Index under the word* "Want," *with the other Papers of that gentleman, but will be found under the Article* "Colchicum."

*We are much obliged to two gentlemen who have sent us* Reviews. *It must, however, be understood that such communications can only be used as the means of assisting our enquiries.*

---

*Our Correspondents will please to be particular in the Address of their Communications, which should be thus,* "To the EDITORS of the LONDON MEDICAL and PHYSICAL JOURNAL, at Mr. SOUTER's, No. 1, Paternoster-Row; or at Mr. ADLARD's, 23, Bartholomew-Close."

